# SRSF1 Facilitates Cytosolic DNA-Induced Production of Type I Interferons Recognized by RIG-I

**DOI:** 10.1371/journal.pone.0115354

**Published:** 2015-02-06

**Authors:** Feng Xue, Xia Li, Xiaoqing Zhao, Lanqi Wang, Min Liu, Ruofei Shi, Jie Zheng

**Affiliations:** 1 Laboratory of Dermatology, Ruijin Hospital, School of Medicine, Shanghai Jiao Tong University, Shanghai, China; 2 Department of Dermatology, Ruijin Hospital, School of Medicine, Shanghai Jiao Tong University, Shanghai, China; University of Hong Kong, HONG KONG

## Abstract

**Background:**

Evidence has shown that psoriasis is closely associated with infection; however, the mechanism of this association remains unclear. In mammalian cells, viral or bacterial infection is accompanied by the release of cytosolic DNA, which in turn triggers the production of type-I interferons (IFNs). Type I IFNs and their associated genes are significantly upregulated in psoriatic lesions. RIG-I is also highly upregulated in psoriatic lesions and is responsible for IFN production. However, RIG-I mediated regulatory signaling in psoriasis is poorly understood.

**Methods:**

We screened a cDNA library and identified potential RIG-I interacting partners that may play a role in psoriasis.

**Results:**

We found that serine/arginine-rich splicing factor 1 (SRSF1) could specifically interact with RIG-I to facilitate RIG-I mediated production of type-I IFN that is triggered by cytosolic DNA. We found SRSF1 associates with RNA polymerase III and RIG-I in a DNA-dependent manner. In addition, treatment with a TNFα inhibitor downregulated SRSF1 expression in peripheral blood mononuclear cells (PBMCs) from psoriasis vulgaris patients.

**Discussion:**

Based on the abundance of pathogenic cytosolic DNA that is detected in psoriatic lesions, our finding that RIG-I interacts with SRSF1 to regulate type-I IFN production reveals a critical link regarding how cytosolic DNA specifically activates aberrant IFN expression. These data may provide new therapeutic targets for the treatment of psoriasis.

## Introduction

Psoriasis is a chronic inflammatory skin disease that is currently recognized as a complex immune disorder involving both innate and adaptive immune regulation [1,2]. Studies have shown that only individuals who carry specific genetic susceptible alleles to psoriasis will develop the disease under in certain environments. Among these environmental factors, infection is considered a major contributor to the disease. The primary consequence of bacterial or viral skin infection is abundant cytosolic DNA production, which is a key trigger of the immune response. It has been known since the early 20^th^ century that nucleic acids boost the immune response, which is the basis of some vaccine designs. Physiologically, DNA is stored in the nucleus and mitochondria but is absent from the cytosol or extracellular space. In psoriatic lesions, DNA fragments are abundant in the cytosol [3]. These cytosolic DNAs will be recognized by a variety of DNA sensors that trigger immune activation, releasing proinflammatory cytokines such as interleukins, interferons (IFNs), and TNF. Indeed, our previous work showed streptococcal antigen (SA) without nucleic acid decreases proliferation whereas streptococcal DNA profoundly enhances PBMC proliferation and activation in patients with psoriasis [4], suggesting a critical pathogenic role of cytosolic DNA-triggered pathways in psoriasis.

Mammalian sensors of nucleic acid in the cytosol were only recently discovered [5]. Previously, Toll-like receptors (TLRs) were found to be pathogen recognition receptors that sense DNA and RNA molecules [6]. However, it was reported that in the absence of TLR signaling, cells remained capable of responding to double-strand DNA stimulation [7]. In the past decade, the identification of host non-TLR receptors that recognize pathogen-derived nucleic acids has revealed an essential role for nucleic acid sensing in immunity initiation. These include DAI (DNA-dependent activator of interferon-regulatory factors) [8], AIM2 (absent in melanoma 2) [9–12], RNA polymerase III [13,14], LRRFIP1 (leucine-rich repeat interacting protein-1 [15], IFI16 (the IFN-inducible protein) [16], DDX41 [17], DHX9, and DHX36 [18]. Very recently, another cytosolic DNA sensor, cGAMP synthase (cGAS), was reported to directly bind DNA and catalyze cGAMP synthesis, which could activate the STING pathway to transcribe type I IFNs [19,20]. Some DNA sensors, such as AIM2, will activate the inflammasome pathway and caspase-1 to cleave pro-IL-1β and release IL-1β, which is critical in cutaneous inflammation [9–12]. However, most of these sensors activate the type-I IFN pathway upon double-strand DNA stimulation. In psoriatic skin, robust overexpression of type I IFN—inducible genes was found [21–23]. It is not fully understood which sensor is responsible in psoriasis. Interestingly, immunohistochemistry studies revealed high levels of RIG-I expression in the epidermal cells and macrophages infiltrating the psoriatic lesions but not in normal epidermal cells[24]. Although RIG-I is a double-strand RNA sensor [25,26], its signaling can be triggered by RNAs transcribed from Pol III, a cytosolic DNA sensor [14]. Because RIG-I is highly expressed in psoriasis lesions and macrophages, we hypothesized that RIG-I (or its partners) may play an important role in the initiation and progression of the disease. In addition, RIG-I is regulated by ubiquitination; for example, K48-linked ubiquitin chains may target RIG-I for degradation whereas K63-linked ubiquitin chains stabilize it and activate signaling cascades. We used a commercially available cDNA library to screen for proteins that interact with RIG-I, including ubiquitin-specific protease (USP) family members. We found that USP3 and a splicing-factor oncoprotein, serine/arginine-rich splicing factor 1 (SRSF1), interact with RIG-I. Because USP3 regulates RIG-I activity[27], we focused on whether SRSF1 regulates RIG-I activity. We found that SRSF1 specifically facilitates cytosolic DNA-triggered type I IFN production by association with the RIG-I/RNA polymerase III complex. Notably, SRSF1 expression is downregulated in PBMCs from patients treated for psoriasis.

## Materials and Methods

### cDNA library

Genome-scale Mammalian Gene Collection human cDNA libraries were purchased from Thermo Scientific (MMM5644). Clones were selected and cloned into Flag- or HA-tagged pcDNA 3.1 vectors.

### Reagents and Antibodies

RNA polymerase subunit C32 and SRSF1 antibodies were purchased from Santa Cruz Biotechnology: Pol III RPC32 antibody (H-9): sc-48365, SF2/ASF Antibody (P-15): sc-10254. Poly (dA:dT)/LyoVec, poly (I:C)/LyoVec, and 5′ppp-dsRNA were purchased from Invivogen. Human IFN-β ELISA kits were obtained from PBL Interferon Source. Human IL-6, TNFα, and IL-1β ELISA Duoset were purchased from R&D Systems. ML-60218 was purchased from SYMANSIS.

### Coimmunoprecipitation assays

Cells were lysed in low-salt lysis buffer in the presence of a protease inhibitor. The lysis must be mild so it does not interfere with antibody-antigen binding, but must efficiently extract proteins from the cytoplasm. Lysates were pre-cleared for 1 h at 4°C using protein-G beads. The pre-clearing step reduces background due to adhesion of sticky sample components to the beads. Antibodies pre-bound to beads (anti-FLAG/anti-HA) or antibodies in combination with protein-G beads were added and incubated overnight at 4°C with slow rotation. After antibody-bead complex binding, beads were washed 5 times in washing buffer containing mild denaturants that break nonspecific interactions. Proteins were eluted by boiling the beads in reducing SDS-sample loading buffer. For coimmunoprecipitation experiments, high-sensitivity western blotting substrates were used for protein detection.

### Luciferase reporter assays

HEK293T cells were transfected with *ISRE* luciferase, pRL-TK Renilla luciferase, and different expression or control vectors using Lipofectamine 2000 (Invitrogen). Poly (I:C) (1 μg/mL), poly(dA:dT) (200 ng/mL), and exogenous RIG-I plasmids were used as stimulators. Luciferase activity was measured using a dual luciferase assay kit (Promega) and a Luminoskan Ascent luminometer (Thermo Scientific).

### Patient treatments and RNA isolation from PBMCs

This study was performed in accordance with the Declaration of Helsinki and was approved by the Research Ethics Committee of Shanghai Rui Jin Hospital. All participants provided written informed consent. Psoriasis patients were treated once a week with 40 mg of the TNFα inhibitor adalimumab (Humira from AbbVie) at weeks 1, 3, 5, 7, 9, and 11, and with 80 mg at weeks 0 and 2. PBMCs from patients with psoriasis treated with adalimumab at week 0 and 12 were extracted and isolated by Ficoll gradient centrifuge. PBMCs were washed twice with PBS and lysed using Trizol reagent (Invitrogen). RNA isolation was performed using RNeasy mini kits from Invitrogen according to the manufacturer’s instructions.

### Real-time PCR Analyses

First-strand cDNA was generated from total RNA using oligo-dT and reverse transcriptase (Takara). Real-time PCR was conducted using QuantiTect SYBR Green PCR Master Mix (QIAGEN) with specific primers on an ABI Prism 7000 analyzer (Applied Biosystems). The following primers were used: hSRSF1: forward 5′- CCGCAGGGAACAACGATTG-3′, reverse 5′ GCCGTATTTGTAGAACACGTCCT-3′; hGAPDH: forward 5′- GGTCGGAGTCAACGGATTTGG-3′, reverse 5′-CATGGAATTTGCCATGGGTGGAATC-3′.

### SRSF1 knockdown using RNAi and shRNA

*SRSF1*-specific and control (two-scramble mix) siRNA oligonucleotides were purchased from Invitrogen, and transfected into human PBMCs using Nucleofector kits (Amaxa) according to the manufacturer’s instructions. *SRSF1* shRNAs were purchased from Open Biosystems. Lentiviral-based constructs were transfected into cells using Lipofectamine 2000 (Invitrogen). For shRNA knockdown, 293T cells (in 24-well plates) were transfected with 500 ng shRNAs (non-silencing or *SRSF1*-specific) using Lipofectamine 2000. Twenty-four hours post-transfection, cells were split and reseeded in 24 well plates (2 × 10^5^ cells/mL) to transfect plasmids (such as RIG-I). For siRNA transfection, *SRSF1* siRNA and scrambled control siRNA (300 pmol) were electroporated on day 0. Cells were cultured for 36 h before stimulation. Poly(dA:dT)/LyoVec (1 μg/mL) was used to stimulate human PBMCs and THP-1 cells for another 24 h. Supernatants were collected for ELISA.

### Statistical Analyses

Data are reported as the mean ± standard error of the mean (SEM) of three independent experiments. Comparisons between groups were performed using two-tailed paired Student’s t tests. Asterisks indicate significant differences between groups (*p < 0.05 or **p < 0.01 as determined by Student’s t tests).

## Results

### SRSF1 specifically interacts with RIG-I

**Figure 1:**
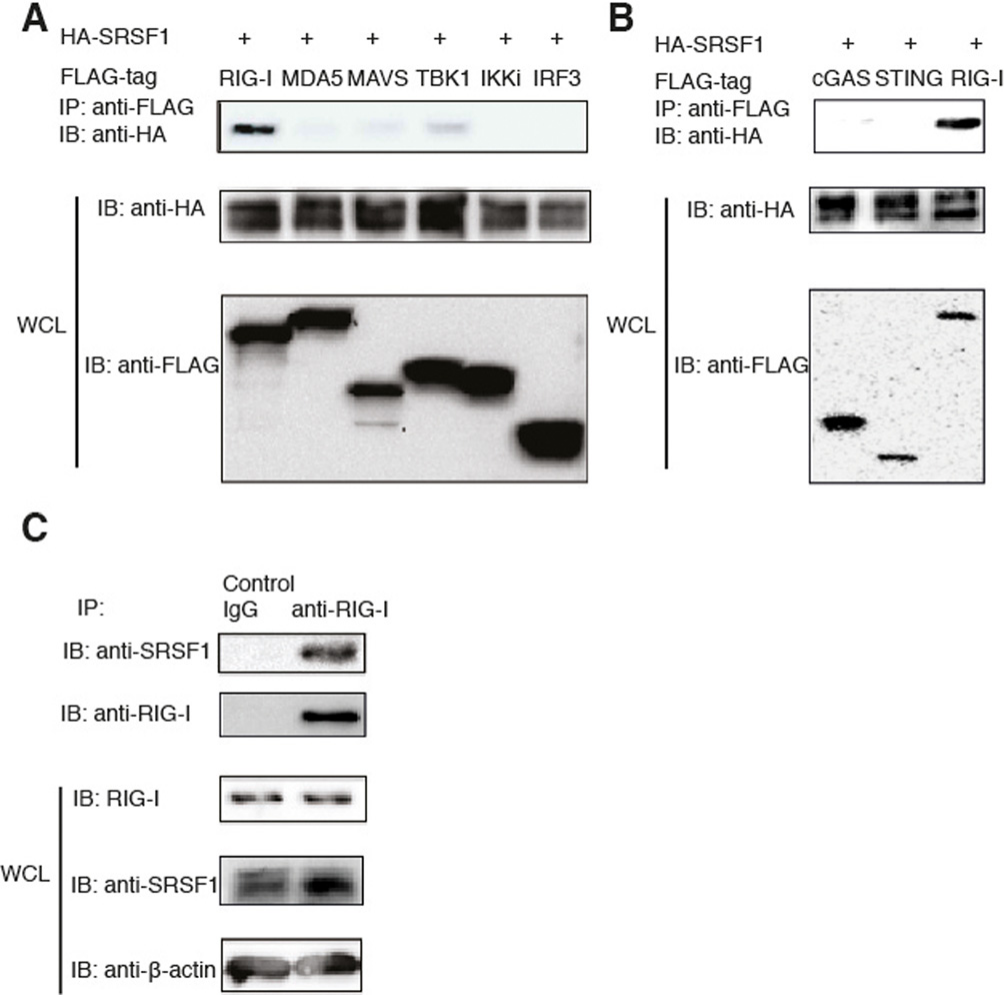
SRSF1 interacts with RIG-I *in vitro* and *in vivo*. A. HEK293T cells were transfected with Flag-RIG-I, Flag-MDA5, Flag-MAVS, FLAG-TBK1, Flag-IKKi, Flag-IRF3, and HA-SRSF1. Flag-tagged proteins were immunoprecipitated using anti-Flag beads and immunoblotted with the HA antibody. B. HEK293T cells were transfected with Flag-tagged cGAS, STING, or RIG-I and HA-tagged SRSF1. Flag-tagged proteins were immunoprecipitated using anti-Flag beads and immunoblotted with the anti-HA antibody. C. THP-1 cells were lysed in low-salt lysis buffer. Cell lysates were immunoprecipitated with the control antibody or the anti-RIG-I antibody, and incubated overnight with protein (A+G). Immunoprecipitated products were immunoblotted with the anti-SRSF1 antibody. WCL, whole cell lysate.

To determine which genes play a critical role in regulating the RIG-I-mediated type-I IFN pathway, we cloned randomly selected genes from a cDNA library (Thermo Scientific) with a Flag or HA. These genes predominantly belong to the USP protein family (approximately 60 proteins termed ubiquitin-specific proteases). USPs regulate many cellular processes by controlling the length of protein ubiquitin chains attached to the target protein. To examine which proteins directly interact with RIG-I, we transfected 293T cells with HA-tagged candidate genes together with Flag-tagged RIG-I. Coimmunoprecipitation and western blot analyses revealed that USP3 and SRSF1 specifically interact with RIG-I *in vitro* (Fig. 1A and S1 Fig.). Because it was recently shown that USP3 interacts with RIG-I to deubiquitinate K63-linked poly-ubiquitin chains on RIG-I and inhibit type I IFN pathways [27], we focused on SRSF1 for further experiments. Recently, a novel STING-mediated DNA sensing pathway was recognized [28]. However, we did not observe any interactions between cGAS or STING and SRSF1 after transfecting 293T cells with Flag-tagged cGAS or STING and HA-tagged SRSF1 and performing coimmunoprecipitation experiments (Fig. 1B). To further determine the specificity of the SRSF1 interaction with RIG-I, we transfected 293T cells with HA-tagged SRSF1 together with Flag-tagged RIG-I, MDA5, MAVS, TBK1, IKKi, and IRF3. When these genes were overexpressed in 293T cells, we observed very weak interactions between SRSF1 and MAVS or TBK1 (Fig. 1A). Next, we used immune cells to determine endogenous interactions. We confirmed that SRSF1 interacts with RIG-I in the human macrophage cell line THP-1 (Fig. 1C). However, SRSF1 did not interact with either MAVS or TBK1 in THP-1 cells (data not shown). These results indicate that SRSF1 specifically interacts with RIG-I under physiological conditions.

### SRSF1 enhances cytosolic DNA-mediated activation of type-I IFN pathways

**Figure 2:**
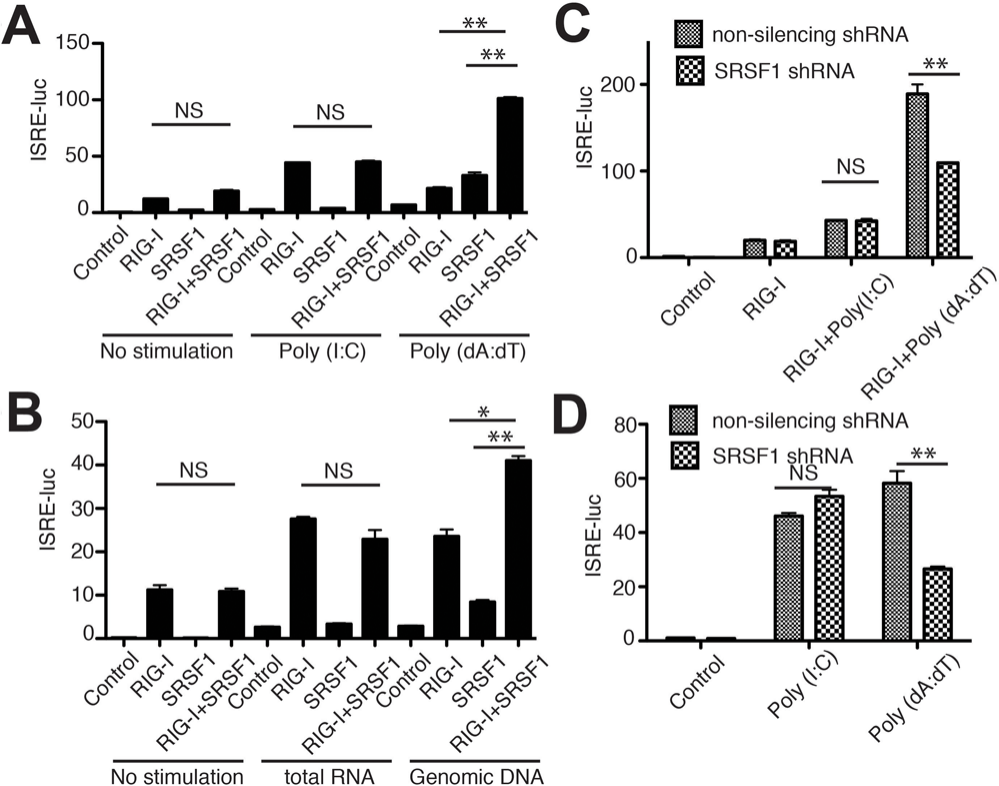
SRSF1 facilitates cytosolic DNA-mediated activation of RIG-I. (A, B) HEK293T cells were transfected with the indicated plasmids, along with ISRE-luc. Twenty-four hours after transfection, cells were stimulated for 12 h with poly(I:C)/LyoVec or poly(dA:dT)/LyoVec (A) or total 293T RNA/salmon sperm DNA (B). (C) *SRSF1* was knocked down and RIG-I was overexpressed in 293T cells. ISRE-luciferase activity was determined after poly(I:C)/LyoVec or poly(dA:dT)/LyoVec treatment. (D) *SRSF1* was knocked down in 293T cells. ISRE-luciferase activity was determined after poly(I:C)/LyoVec or poly(dA:dT)/LyoVec treatment.

To investigate the role of SRSF1 in RIG-I-mediated activation of type-I IFN pathways, we utilized a dual luciferase reporter system. We found that when the synthetic RNA poly (I:C)/LyoVec was stimulated, SRSF1 overexpression did not influence RIG-I mediated activation of ISRE reporter activity. In contrast, overexpression of SRSF1 significantly enhanced poly(dA:dT)/LyoVec stimulated type-I IFN pathway activation (Fig. 2A).

Poly(I:C) is a synthetic analog of double-stranded RNA (dsRNA) whereas poly (dA:dT) is a repetitive synthetic double-stranded DNA sequence and a synthetic analog of B-DNA [poly(I:C)/LyoVec is herein abbreviated poly(I:C) and poly(dA:dT)/LyoVec is abbreviated poly (dA:dT)]. Next, we confirmed these results using total RNA from 293T cells or sonicated salmon sperm DNA and found that SRSF1 specifically enhanced DNA-triggered signaling cascades leading to ISRE luciferase activity (Fig. 2B). Next, we used shRNA against *SRSF1* to validate its physiological role in RIG-I mediated type-I IFN pathways. S2 Fig. shows efficient SRSF1 knockdown by shRNA transfection. Consistent with the results obtained from the overexpression experiments, *SRSF1* knockdown in 293T cells significantly reduced the ISRE luciferase activity mediated by poly (dA:dT) and RIG-I (Fig. 2C). Furthermore, *SRSF1* knockdown impaired endogenous RIG-I mediated type-I IFN activation triggered by poly(dA:dT) but not by poly(I:C) (Fig. 2D). Notably, endogenous SRSF1 expression is upregulated by ligand stimulation, particularly poly(dA:dT) stimulation (S2 Fig.). It remains unknown whether TLR/RLR signaling pathways trigger SRSF1 expression. However, these results indicate that SRSF1 specifically enhanced cytosolic DNA-, but not RNA-, triggered activation of RIG-I-mediated pathways.

### SRSF1 associates with RNA polymerase III and RIG-I in a DNA-dependent manner

**Figure 3:**
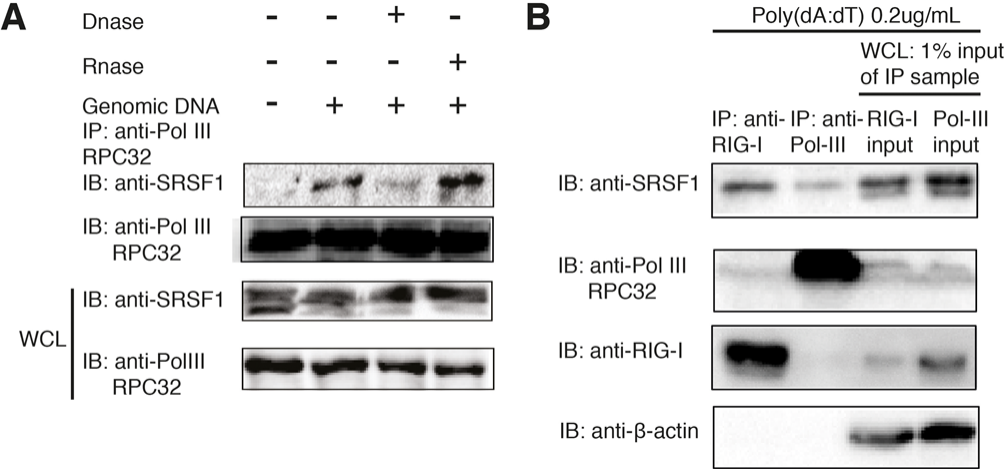
The association of SRSF1 with RNA polymerase III is dependent on the DNA template. (A) HEK293T cells were transfected with salmon sperm DNA and lysed using RIPA buffer. Cell lysates were immunoprecipitated with the RNA polymerase III subunit C7 antibody and immunoblotted using the SRSF1 antibody. Lysates were left untreated or were treated for 1 h with RNase A or DNase I before immunoprecipitation. (B) HEK293T cells were stimulated with 500 ng Poly(dA:dT)/LyoVec. After 24 h, cell lysates were immunoprecipitated with the anti-RIG-I antibody or anti-Pol-III RPC32 antibody and incubated overnight with protein(A+G). Immunoprecipitated products, as well as 1% input from whole cell lysates, were immunoblotted with anti-SRSF1, anti-RIG-I, and anti-Pol III antibodies.

It has been reported that DNA is only sensed by RIG-I when it is reverse transcribed by RNA polymerase III [14]. Therefore, we hypothesized that SRSF1 could facilitate this process and associate with RNA polymerase III. We tested this hypothesis using coimmunoprecipitation assays and found that SRSF1 does not interact with RNA polymerase III until DNA fragments are added to stimulate the cells. In addition, we also observed that treatment of cell lysate with DNase I, but not RNase A, can abrogate this interaction (Fig. 3A). We also noted that after DNA stimulation, SRSF1 is predominantly detected in an upper band (Fig. 3A). We hypothesized that the upper band portion is the protein that was associated with RIG-I or RNA polymerase III. Therefore, we immunoprecipitated endogenous RIG-I and RNA polymerase III in poly(dA:dT)-treated 293T cells and compared the immunoprecipitated protein with the whole cell lysate sample (input). Indeed, we found that the upper band of SRSF1 was interacting with RIG-I and Pol-III, whereas the lower band was not (Fig. 3B). Interestingly, RIG-I and RNA polymerase III do not directly interact because a very weak band is detected in the fraction from the anti-RIG-I immunoprecipitated lysate and no band is detected in the fraction from the anti-Pol III immunoprecipitated lysate (Fig. 3B). These results suggest that SRSF1 is a bridge between RIG-I and Pol-III. Next, to test whether enhanced type-I IFN production by SRSF1 is dependent on the RNA polymerase III pathway, we used a small molecule inhibitor of RNA polymerase III, ML-60218, in HEK293T cells. We then transfected cells with poly(dA:dT) or poly(I:C) or 5′ triphosphate double-stranded RNA. Cells were also transfected with SRSF1 and ISRE-luciferase reporter plasmids. ML-60218 strongly inhibited luciferase activity in poly(dA:dT)-transfected cells but not in poly(I:C) or 5′ triphosphate double-stranded RNA transfected cells (S3 Fig.). Because poly(I:C) and 5′ triphosphate double-stranded RNA can directly activate RIG-I independent of RNA polymerase III, we conclude that the effect of SRSF1 in activating the type I IFN pathway depends RNA polymerase III activity. Taken together, these results explain the molecular machinery regarding how SRSF1 facilitates DNA sensing in a RNA polymerase III-RIG-I mediated pathway.

### SRSF1 is downregulated in psoriasis patients after treatment and SRSF1 knockdown decreases type I IFN production

**Figure 4:**
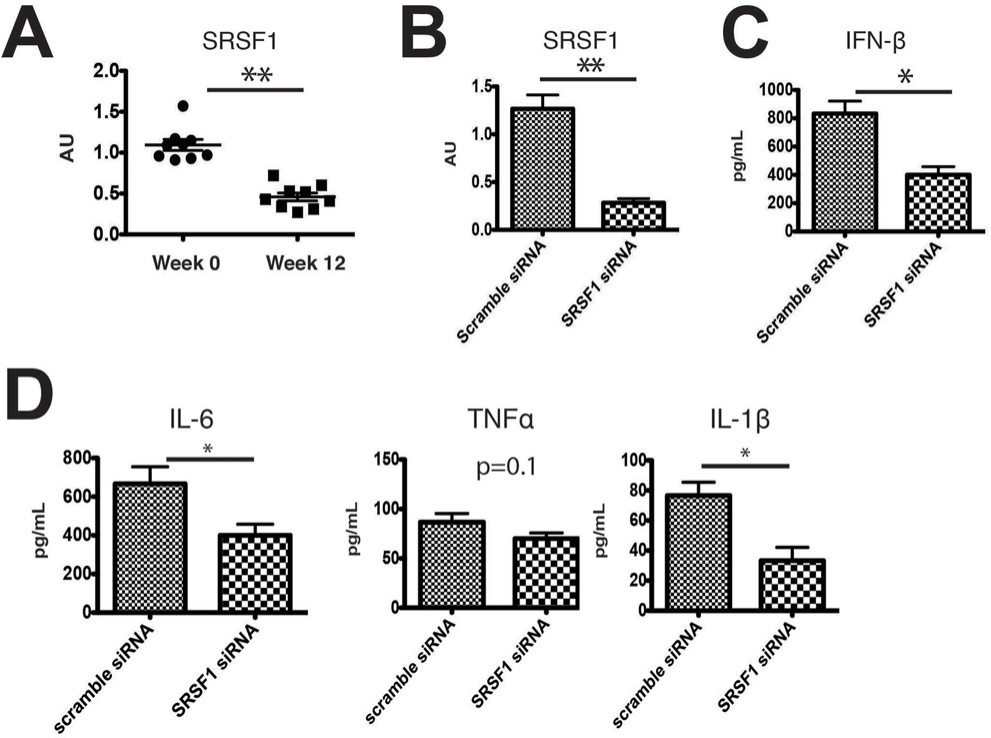
Downregulated SRSF1 in psoriasis patients after treatment and *SRSF1* knockdown decreases type I IFN production. A. *SRSF1* mRNA levels in PBMCs from patients with psoriasis at week 0 or after 12 weeks of treatment with adalimumab. B. Specific *SRSF1* knockdown was evaluated in PBMCs transfected with *SRSF1* siRNA or scrambled siRNA. PBMC samples are from two psoriasis patients before drug treatment. Cells were combined and electroporated with scrambled siRNA or *SRSF1* siRNA. C. IFN-β concentration in the supernatant of samples stimulated for 24 h with 1 μg/mL poly(dA:dT)/LyoVec for 24hrs. D. IL-6, TNFα, and IL-1β concentration in the supernatant of samples stimulated for 24 h with 1 μg/mL poly(dA:dT)/LyoVec.

Finally, we sought to determine if SRSF1 has a role in psoriasis. Because SRSF1 was reported to be upregulated in various tumors [29–31], we hypothesized that it might be upregulated by the pro-survival and inflammatory transcription factor NF-κB. In psoriasis patients, TNF-α activates NF-κB, and TNF-α or TNF-α receptor inhibitors are very effective in suppressing inflammation. To test whether inhibition of TNF-α could suppress SRSF1 expression in patients with psoriasis, we collected PBMCs from patients at weeks 0 and 12 after treatment with adalimumab. As shown in S4A and S4B Fig., adalimumab treatment over a 12 week period significantly alleviated disease severity. Hyperkeratosis, absence of the granular layer, and epidermal hyperplasia improved significantly as shown by histopathological sections (S4C Fig.). The therapeutic effects of adalimumab were evaluated by PASI scores and were significantly different in the nine patients who agreed to therapy (S4D Fig.). We found that SRSF1 levels in the PBMCs from these nine patients significantly decreased after treatment for 12 weeks (Fig. 4A). Next, to determine what ligand stimuli could induce potent type I IFN production in psoriasis patients by RIG-I dependent manner, we isolated three psoriasis patients’ PBMCs and stimulated them with one of the four ligands: poly(dA:dT), 5′ppp-dsRNA, plasmid DNAs isolated from E.coli with EndoFree plasmid kits or sonicated salmon sperm DNAs. In the meantime, we transfected siRNAs against either *RIG-I* or *cGAS* or scrambled siRNA as control to determine which pathway is most responsible for these stimulation as well. The supernatants were collected after 24 h after ligand stimulation, the concentration of IFN-β in the supernatant was measured using ELISA. As shown in S5 Fig., we found that poly (dA:dT) induced the most profound production of IFN-β in patients’ PBMCs among the DNA ligands to levels comparable to a direct RIG-I ligand, 5′ppp-dsRNA. In addition, we found that poly (dA:dT) induced type-I IFN production is dependent on RIG-I but not cGAS in psoriasis patients’ PBMCs. Interestingly, sensing of plasmid DNAs isolated from E.coli depends on cGAS but not RIG-I. Why different DNA types induces different pathway is not fully understood, but compare to E.coli DNAs, poly(dA:dT) is a much more potent ligand. Thus, we set out to determine whether SRSF1 expression contributes to type-I IFN production in poly(dA:dT) stimulated PBMCs. Briefly, PBMCs from these patients were knocked down *SRSF1* gene expression by electroporation with *SRSF1* or scrambled siRNAs. We achieved approximately 80% knockdown efficacy using electroporation kits (Fig. 4B). We then stimulated PBMCs transfected with either scrambled or *SRSF1* siRNAs with poly(dA:dT). After 24 h, the concentration of IFN-β in the supernatant was measured using ELISA. We found that IFN-β production was significantly lower when SRSF1 expression was compromised (Fig. 4C). To investigate what sub-population is responsible for SRSF1-mediated IFN-β production, we added the siRNAs to the human macrophage cell line THP-1. THP-1 cells were electroporated with either scrambled siRNAs or siRNAs targeting human *SRSF1*. After 36 h, cells were stimulated with 1 μg/mL poly(dA:dT) and subjected to RT-PCR analyses. The *SRSF1* knockdown efficiency in THP-1 cells was approximately 80% (S6A Fig.). ELISA analyses revealed significantly decreased IFN-β in the supernatants when SRSF1 expression was knocked down (S6B Fig.). In addition, we also evaluated the concentration of other pro-inflammatory cytokines, including IL-6, TNFα, and IL-1β in human PBMCs. We found that except for TNFα, the levels of these cytokine were significantly decreased after *SRSF1* knockdown (*p < 0.05 or **p < 0.01). We conclude that *SRSF1* knockdown results in fewer RNA ligands transcribed by RNA polymerase III from poly(dA:dT) and leads to sub-optimal activation of RIG-I and inflammatory cytokine production.

## Discussion

Activation of pattern recognition receptors (PRRs) by their corresponding ligands, pathogen associated molecular patterns (PAMPs), initiates several critical signaling pathways that lead to the production of proinflammatory cytokines such as TNF-α and type-I IFNs. These proinflammatory cytokines in turn induce profound positive feedback for adaptive immune responses [32–34]. Increasing evidence shows that many autoimmune diseases likely result from dysregulated innate immunity [35–37]. Thus, understanding the key regulators of innate immunity signaling should help control inflammation-induced autoimmune diseases, including psoriasis. Due to a close relationship between infection and psoriasis, it is important to identify any links that exist. With the development of genomic sequencing technology, genome-wide associated studies (GWAS) have revealed a possible candidate connecting infection to psoriasis.

Several recently identified single-nucleotide polymorphisms (SNPs) that are linked to psoriasis susceptibility are found adjacent or in close proximity to genes associated with the innate immune response, such as IFIH1 (MDA5), NFKBIA, STAT3, and SOCS1 [38]. It is noteworthy that recent GWAS have identified 15 new psoriasis susceptibility loci that are highly related to innate immunity regulation [39]. Among the new loci, DDX58 encodes human RIG-I receptor that recognizes cytosolic RNA, which plays a crucial role in psoriasis [39]. RIG-I cannot directly bind bacterial DNA; however, it could indirectly recognize viral RNA that is transcribed by RNA polymerase III in cells [14]. Here, we demonstrate SRSF1 is a critical bridge between RIG-I and RNA polymerase III in cytosolic DNA sensing. Another recently discovered cytosolic DNA pathway is mediated by STING. The upstream DNA sensors involved in the STING pathway include C gas, IFI16, and DDX41 [16,17,40–42]. Although we did not observe an interaction between SRSF1 and STING, further investigations are required to determine whether SRSF1 is involved in the recognition of DNA by cGAS, IFI16, or DDX41.

SR proteins are a family of proteins involved in RNA splicing [43] and RNA metabolism [44], including chromatin remodeling, transcription, nonsense-mediated messenger RNA (mRNA) decay (NMD), and mRNA export and stability [45–47]. Recent findings found that SR proteins bind to genome-wide DNA promoter regions, introns, exons, and intragenic regions [48]. In particular, SRSF2 can release paused RNA polymerase II enzymes from binding near the transcription start sites of numerous genes [48]. It is notable that RNA polymerase pause release is now recognized as a crucial step in gene transcription [49]. Interestingly, in our study, we found that SRSF1 facilitates Pol-III dependent recognition of cytosolic DNA by RIG-I. It is unknown whether RNA polymerase III pauses during transcription. However, we hypothesize that similarities in transcription between RNA polymerase II and III may exist. If this is the case, it is likely that SRSF1 utilizes a similar mechanism as SRSF2/Pol II to promote intracellular DNA transcription. Further studies are required to determine the detailed mechanisms of this regulation.

SRSF1 is considered a proto-oncogene that is overexpressed in various cancers [29–31]. The oncogenic properties of SRSF1 are mediated in part via alternate splicing of various oncogenes and tumor suppressors [29,50] as well as by p53 [51]. It is noteworthy that there are significantly more p53-positive cells in the skin of psoriatic patients [52], which may be stabilized by SRSF1. In addition, SRSF1 is reported to participate in metabolic pathways, including the mTOR pathway [53]. Altered mTOR signaling in the epidermis may lead to the psoriatic phenotype [54]. Taken together, these results suggest it is highly probable that SRSF1 plays a crucial role in psoriasis pathogenesis in various ways (via RIG-I, p53, and mTOR). Therefore, SRSF1 presents an opportunity for future psoriasis targeted therapies. SRSF1 has not been associated with immune regulation until recently. A recent report showed that SRSF1 enhances IL-2 production in T cells from systemic lupus erythematosus (SLE) patients [55]. Our study indicates that SRSF1 bridges with RIG-I and RNA polymerase III in type I IFN signaling and is another example of its unrecognized role in regulating the immune system.

It is noteworthy that immunoblots for SRSF1 in an overexpression system or endogenous system often elicits two distinct bands. After cells were activated by poly(dA:dT), the upper band became the dominant band (Fig. 3A). We speculate that SRSF1 can be modified into an active form in which the molecular weight is slightly higher. This modification is likely phosphorylation under certain cellular stresses [56]. It is unclear whether facilitation of the RIG-I-mediated type I IFN pathway is due to phosphorylated SRSF1. However, in our coimmunoprecipitation assays, we found that only the upper band can be immunoprecipitated with RIG-I or Pol-III (Fig. 3B). If the active form of SRSF1 is phosphorylated, a drug screen can be performed with the phosphorylation site as a drug target that will specifically inhibit its role in promoting inflammation mediated by the RIG-I pathway and alleviate disease severity.

Finally, we found that SRSF1 levels were downregulated after patients with psoriasis were treated with anti-inflammatory agents, including a TNFα inhibitor. Xiong et al. [57] reported that SRSF1 is downregulated in inflammatory myositis due to TNFα stimulation. Although SRSF1 was trivially downregulated in muscle biopsy samples from inflammatory myositic patients, it is very likely that SRSF1 regulation is cell-type specific. The transcriptional factors controlling SRSF1 expression may differ dramatically between muscle cells and blood cells. We used cytokines to stimulate THP-1 cells and determined that no single cytokine enhances SRSF1 expression. In 293T cells, we found that poly(dA:dT) treatment enhanced SRSF1 expression (S2 Fig.). Therefore, we evaluated SRSF1 expression in skin lesions or non-lesions. SRSF1 expression in skin lesions varies greatly between individuals (data not shown). Whether application of antibacterial agents would decrease SRSF1 expression in skin lesions from patients is currently being investigated. Further studies on SRSF1 transcriptional regulation will be valuable to determine if SRSF1 is a potential target therapy for psoriasis. Although SRSF1 is downregulated in PBMCs after treatment with adalimumab, we did not observe significant differences in SRSF1 expression in PBMCs between psoriasis patients and healthy donors (data not shown). This result suggests that SRSF1 alone does not cause psoriasis initiation; however, it could be an essential factor for cytosolic DNA sensing in normal conditions. These data are consistent with GWAS results that indicate that SRSF1 is not a genetically susceptible allele for psoriasis but RIG-I plays a major role in disease initiation [39]. Based on our current results, temporary ablation of SRSF1 may be beneficial for ameliorating disease severity and the production of inflammatory cytokines.

## Supporting Information

S1 Fig Screen for RIG-I interacting proteins. USP family proteins and SRSF1, ATG5, and ATG101 were cloned into pcDNA-HA vectors and cotransfected with Flag-tagged RIG-I into HEK293T cells. Immunoprecipitations were performed using anti-HA beads and immunoblotting was performed using the anti-Flag antibody.
(TIF)

S2 Fig SRSF1 and RIG-I expression in knockdown samples. Immunoblot of the samples used in the luciferase assays shown in Fig. 2C using the anti-Flag and anti-SRSF1 antibodies. Actin served as a loading control.
(TIF)

S3 Fig SRSF1 facilitates ISRE-luciferase activity that is dependent on RNA-polymerase III. HEK293T cells were treated with ML-60218 (30 μM) or DMSO for 2 h. Cells were subsequently transfected with 0.2 μg/mL poly(dA:dT), 1 μg/mL poly(I:C), or 1 μg/mL 5′ triphosphate double-stranded RNA in conjunction with SRSF1 and ISRE-luciferase reporter plasmids. Luciferase activity was analyzed 24 h after transfection.
(TIF)

S4 Fig Improvement in psoriasis patients who were treated with the TNF-α inhibitor adalimumab for 12 weeks. (A, B) Two patients with typical moderate-to-severe chronic plaque psoriasis before and after treatment for 12 weeks with adalimumab are shown.
(C) Simultaneous transformations could be observed in patient histopathology. Hyperkeratosis, absence of the granular layer, and epidermal hyperplasia improved after 12 weeks of adalimumab treatment.
(D) PASI scores of the nine enrolled patients before and after adalimumab treatment.
(TIF)

S5 Fig poly (dA:dT) but not E.coli DNA sensing is dependent on RIG-I in psoriasis patients. IFN-β concentration in the supernatant of samples stimulated for 24 h with 1 μg/mL poly(dA:dT)/LyoVec or 1ug/mL 5’ppp-dsRNA or 1ug/mL plasmid DNA extracted from E.coli or 1ug/mL sonicated salmon sperm DNAs for 24hrs. Before stimulation, PBMCs from three psoriasis patients before drug treatment were transfected with 300 pmol human *RIG-I* or *cGAS* stealth siRNA or scrambled siRNA from invitrogen by electrophoresis and rest for 12 h.
(TIF)

S6 Fig *SRSF1* knockdown in THP-1 cells reduces IFN-β production. (A) THP-1 cells were electroporated with 300 pmol scrambled siRNA or siRNA targeting human *SRSF1*. Real-time PCR analyses were used to assess the knockdown efficiency of SRSF1.
(B) Thirty-six hours after transfection, THP-1 cells were stimulated for 24 h with 1 μg/mL poly (dA:dT)/LyoVec. Cell supernatants were collected and cytokine levels were measured by ELISA.
(TIF)

## References

[1] Krueger JG (2002) The immunologic basis for the treatment of psoriasis with new biologic agents. Journal of the American Academy of Dermatology 46: 1–23; quiz 23–26. 10.1067/mjd.2002.120568 11756941

[2] Lebwohl M (2003) Psoriasis. Lancet 361: 1197–1204. 10.1016/S0140-6736(03)12954-6 12686053

[3] Dombrowski Y, Peric M, Koglin S, Kammerbauer C, Goss C, et al. (2011) Cytosolic DNA triggers inflammasome activation in keratinocytes in psoriatic lesions. Science translational medicine 3: 82ra38 10.1126/scitranslmed.3002001 21562230 PMC3235683

[4] Cai YH, Lu ZY, Shi RF, Xue F, Chen XY, et al. (2009) Enhanced proliferation and activation of peripheral blood mononuclear cells in patients with psoriasis vulgaris mediated by streptococcal antigen with bacterial DNA. The Journal of investigative dermatology 129: 2653–2660. 10.1038/jid.2009.153 19609313

[5] Yoneyama M, Fujita T (2007) Cytoplasmic double-stranded DNA sensor. Nature immunology 8: 907–908. 10.1038/ni0907-907 17712341

[6] Hemmi H, Takeuchi O, Kawai T, Kaisho T, Sato S, et al. (2000) A Toll-like receptor recognizes bacterial DNA. Nature 408: 740–745. 10.1038/35047123 11130078

[7] Ishii KJ, Coban C, Kato H, Takahashi K, Torii Y, et al. (2006) A Toll-like receptor-independent antiviral response induced by double-stranded B-form DNA. Nature immunology 7: 40–48. 10.1038/ni1282 16286919

[8] Takaoka A, Wang Z, Choi MK, Yanai H, Negishi H, et al. (2007) DAI (DLM-1/ZBP1) is a cytosolic DNA sensor and an activator of innate immune response. Nature 448: 501–505. 10.1038/nature06013 17618271

[9] Burckstummer T, Baumann C, Bluml S, Dixit E, Durnberger G, et al. (2009) An orthogonal proteomic-genomic screen identifies AIM2 as a cytoplasmic DNA sensor for the inflammasome. Nature immunology 10: 266–272. 10.1038/ni.1702 19158679

[10] Fernandes-Alnemri T, Yu JW, Datta P, Wu J, Alnemri ES (2009) AIM2 activates the inflammasome and cell death in response to cytoplasmic DNA. Nature 458: 509–513. 10.1038/nature07710 19158676 PMC2862225

[11] Hornung V, Ablasser A, Charrel-Dennis M, Bauernfeind F, Horvath G, et al. (2009) AIM2 recognizes cytosolic dsDNA and forms a caspase-1-activating inflammasome with ASC. Nature 458: 514–518. 10.1038/nature07725 19158675 PMC2726264

[12] Roberts TL, Idris A, Dunn JA, Kelly GM, Burnton CM, et al. (2009) HIN-200 proteins regulate caspase activation in response to foreign cytoplasmic DNA. Science 323: 1057–1060. 10.1126/science.1169841 19131592

[13] Ablasser A, Bauernfeind F, Hartmann G, Latz E, Fitzgerald KA, et al. (2009) RIG-I-dependent sensing of poly(dA:dT) through the induction of an RNA polymerase III-transcribed RNA intermediate. Nature immunology 10: 1065–1072. 10.1038/ni.1779 19609254 PMC3878616

[14] Chiu YH, Macmillan JB, Chen ZJ (2009) RNA polymerase III detects cytosolic DNA and induces type I interferons through the RIG-I pathway. Cell 138: 576–591. 10.1016/j.cell.2009.06.015 19631370 PMC2747301

[15] Yang P, An H, Liu X, Wen M, Zheng Y, et al. (2010) The cytosolic nucleic acid sensor LRRFIP1 mediates the production of type I interferon via a beta-catenin-dependent pathway. Nature immunology 11: 487–494. 10.1038/ni.1876 20453844

[16] Unterholzner L, Keating SE, Baran M, Horan KA, Jensen SB, et al. (2010) IFI16 is an innate immune sensor for intracellular DNA. Nature immunology 11: 997–1004. 10.1038/ni.1932 20890285 PMC3142795

[17] Zhang Z, Yuan B, Bao M, Lu N, Kim T, et al. (2011) The helicase DDX41 senses intracellular DNA mediated by the adaptor STING in dendritic cells. Nature immunology 12: 959–965. 10.1038/ni.2091 21892174 PMC3671854

[18] Kim T, Pazhoor S, Bao M, Zhang Z, Hanabuchi S, et al. (2010) Aspartate-glutamate-alanine-histidine box motif (DEAH)/RNA helicase A helicases sense microbial DNA in human plasmacytoid dendritic cells. Proceedings of the National Academy of Sciences of the United States of America 107: 15181–15186. 10.1073/pnas.1006539107 20696886 PMC2930588

[19] Sun L, Wu J, Du F, Chen X, Chen ZJ (2012) Cyclic GMP-AMP Synthase Is a Cytosolic DNA Sensor That Activates the Type I Interferon Pathway. Science. 10.1126/science.1232458 PMC3863629 23258413

[20] Wu J, Sun L, Chen X, Du F, Shi H, et al. (2012) Cyclic GMP-AMP Is an Endogenous Second Messenger in Innate Immune Signaling by Cytosolic DNA. Science. 10.1126/science.1229963 PMC3855410 23258412

[21] Yao Y, Richman L, Morehouse C, de los Reyes M, Higgs BW, et al. (2008) Type I interferon: potential therapeutic target for psoriasis? PloS one 3: e2737 10.1371/journal.pone.0002737 18648529 PMC2481274

[22] Schmid P, Itin P, Cox D, McMaster GK, Horisberger MA (1994) The type I interferon system is locally activated in psoriatic lesions. Journal of interferon research 14: 229–234. 10.1089/jir.1994.14.229 7861026

[23] van der Fits L, van der Wel LI, Laman JD, Prens EP, Verschuren MC (2004) In psoriasis lesional skin the type I interferon signaling pathway is activated, whereas interferon-alpha sensitivity is unaltered. The Journal of investigative dermatology 122: 51–60. 10.1046/j.0022-202X.2003.22113.x 14962089

[24] Kitamura H, Matsuzaki Y, Kimura K, Nakano H, Imaizumi T, et al. (2007) Cytokine modulation of retinoic acid-inducible gene-I (RIG-I) expression in human epidermal keratinocytes. Journal of dermatological science 45: 127–134. 10.1016/j.jdermsci.2006.11.003 17182220

[25] Yoneyama M, Kikuchi M, Natsukawa T, Shinobu N, Imaizumi T, et al. (2004) The RNA helicase RIG-I has an essential function in double-stranded RNA-induced innate antiviral responses. Nature immunology 5: 730–737. 10.1038/ni1087 15208624

[26] Myong S, Cui S, Cornish PV, Kirchhofer A, Gack MU, et al. (2009) Cytosolic viral sensor RIG-I is a 5′-triphosphate-dependent translocase on double-stranded RNA. Science 323: 1070–1074. 10.1126/science.1168352 19119185 PMC3567915

[27] Cui J, Song Y, Li Y, Zhu Q, Tan P, et al. (2014) USP3 inhibits type I interferon signaling by deubiquitinating RIG-I-like receptors. Cell Res 24: 400–416. 10.1038/cr.2013.170 24366338 PMC3975496

[28] Burdette DL, Monroe KM, Sotelo-Troha K, Iwig JS, Eckert B, et al. (2011) STING is a direct innate immune sensor of cyclic di-GMP. Nature 478: 515–518. 10.1038/nature10429 21947006 PMC3203314

[29] Anczukow O, Rosenberg AZ, Akerman M, Das S, Zhan L, et al. (2012) The splicing factor SRSF1 regulates apoptosis and proliferation to promote mammary epithelial cell transformation. Nature structural & molecular biology 19: 220–228. 10.1038/nsmb.2207 22245967 PMC3272117

[30] Das S, Anczukow O, Akerman M, Krainer AR (2012) Oncogenic splicing factor SRSF1 is a critical transcriptional target of MYC. Cell reports 1: 110–117. 10.1016/j.celrep.2011.12.001 22545246 PMC3334311

[31] Karni R, de Stanchina E, Lowe SW, Sinha R, Mu D, et al. (2007) The gene encoding the splicing factor SF2/ASF is a proto-oncogene. Nature structural & molecular biology 14: 185–193. 10.1038/nsmb1209 17310252 PMC4595851

[32] Akira S, Uematsu S, Takeuchi O (2006) Pathogen recognition and innate immunity. Cell 124: 783–801. 10.1016/j.cell.2006.02.015 16497588

[33] Honda K, Taniguchi T (2006) IRFs: master regulators of signalling by Toll-like receptors and cytosolic pattern-recognition receptors. Nature reviews Immunology 6: 644–658. 10.1038/nri1900 16932750

[34] Kawai T, Akira S (2009) The roles of TLRs, RLRs and NLRs in pathogen recognition. International immunology 21: 317–337. 10.1093/intimm/dxp017 19246554 PMC2721684

[35] Inohara Chamaillard, McDonald C, Nunez G (2005) NOD-LRR proteins: role in host-microbial interactions and inflammatory disease. Annual review of biochemistry 74: 355–383. 10.1146/annurev.biochem.74.082803.133347 15952891

[36] Ting JP, Kastner DL, Hoffman HM (2006) CATERPILLERs, pyrin and hereditary immunological disorders. Nature reviews Immunology 6: 183–195. 10.1038/nri1788 16498449

[37] Tlaskalova-Hogenova H, Tuckova L, Stepankova R, Hudcovic T, Palova-Jelinkova L, et al. (2005) Involvement of innate immunity in the development of inflammatory and autoimmune diseases. Annals of the New York Academy of Sciences 1051: 787–798. 10.1196/annals.1361.122 16127016

[38] Prens EP, Kant M, van Dijk G, van der Wel LI, Mourits S, et al. (2008) IFN-alpha enhances poly-IC responses in human keratinocytes by inducing expression of cytosolic innate RNA receptors: relevance for psoriasis. The Journal of investigative dermatology 128: 932–938. 10.1038/sj.jid.5701087 17928888

[39] Tsoi LC, Spain SL, Knight J, Ellinghaus E, Stuart PE, et al. (2012) Identification of 15 new psoriasis susceptibility loci highlights the role of innate immunity. Nature genetics 44: 1341–1348. 10.1038/ng.2467 23143594 PMC3510312

[40] Parvatiyar K, Zhang Z, Teles RM, Ouyang S, Jiang Y, et al. (2012) The helicase DDX41 recognizes the bacterial secondary messengers cyclic di-GMP and cyclic di-AMP to activate a type I interferon immune response. Nature immunology 13: 1155–1161. 10.1038/ni.2460 23142775 PMC3501571

[41] Ablasser A, Goldeck M, Cavlar T, Deimling T, Witte G, et al. (2013) cGAS produces a 2′-5′-linked cyclic dinucleotide second messenger that activates STING. Nature 498: 380–384. 10.1038/nature12306 23722158 PMC4143541

[42] Xiao TS, Fitzgerald KA (2013) The cGAS-STING Pathway for DNA Sensing. Molecular cell 51: 135–139. 10.1016/j.molcel.2013.07.004 23870141 PMC3782533

[43] Lin S, Fu XD (2007) SR proteins and related factors in alternative splicing. Advances in experimental medicine and biology 623: 107–122. 18380343 10.1007/978-0-387-77374-2_7

[44] Zhong XY, Wang P, Han J, Rosenfeld MG, Fu XD (2009) SR proteins in vertical integration of gene expression from transcription to RNA processing to translation. Molecular cell 35: 1–10. 10.1016/j.molcel.2009.06.016 19595711 PMC2744344

[45] Loomis RJ, Naoe Y, Parker JB, Savic V, Bozovsky MR, et al. (2009) Chromatin binding of SRp20 and ASF/SF2 and dissociation from mitotic chromosomes is modulated by histone H3 serine 10 phosphorylation. Molecular cell 33: 450–461. 10.1016/j.molcel.2009.02.003 19250906 PMC2667802

[46] Michlewski G, Sanford JR, Caceres JF (2008) The splicing factor SF2/ASF regulates translation initiation by enhancing phosphorylation of 4E-BP1. Molecular cell 30: 179–189. 10.1016/j.molcel.2008.03.013 18439897

[47] Zhang Z, Krainer AR (2004) Involvement of SR proteins in mRNA surveillance. Molecular cell 16: 597–607. 10.1016/j.molcel.2004.10.031 15546619

[48] Ji X, Zhou Y, Pandit S, Huang J, Li H, et al. (2013) SR Proteins Collaborate with 7SK and Promoter-Associated Nascent RNA to Release Paused Polymerase. Cell 153: 855–868. 10.1016/j.cell.2013.04.028 23663783 PMC4103662

[49] Adelman K, Lis JT (2012) Promoter-proximal pausing of RNA polymerase II: emerging roles in metazoans. Nature reviews Genetics 13: 720–731. 10.1038/nrg3293 22986266 PMC3552498

[50] Ghigna C, Giordano S, Shen H, Benvenuto F, Castiglioni F, et al. (2005) Cell motility is controlled by SF2/ASF through alternative splicing of the Ron protooncogene. Molecular cell 20: 881–890. 10.1016/j.molcel.2005.10.026 16364913

[51] Fregoso OI, Das S, Akerman M, Krainer AR (2013) Splicing-factor oncoprotein SRSF1 stabilizes p53 via RPL5 and induces cellular senescence. Molecular cell 50: 56–66. 10.1016/j.molcel.2013.02.001 23478443 PMC3628402

[52] Baran W, Szepietowski JC, Szybejko-Machaj G (2005) Expression of p53 protein in psoriasis. Acta dermatovenerologica Alpina, Panonica, et Adriatica 14: 79–83. 16200332

[53] Karni R, Hippo Y, Lowe SW, Krainer AR (2008) The splicing-factor oncoprotein SF2/ASF activates mTORC1. Proceedings of the National Academy of Sciences of the United States of America 105: 15323–15327. 10.1073/pnas.0801376105 18832178 PMC2563124

[54] Buerger C, Malisiewicz B, Eiser A, Hardt K, Boehncke WH (2013) mTOR and its downstream signalling components are activated in psoriatic skin. The British journal of dermatology. 10.1111/bjd.12271 23398394

[55] Moulton VR, Grammatikos AP, Fitzgerald LM, Tsokos GC (2013) Splicing factor SF2/ASF rescues IL-2 production in T cells from systemic lupus erythematosus patients by activating IL-2 transcription. Proc Natl Acad Sci U S A 110: 1845–1850. 10.1073/pnas.1214207110 23319613 PMC3562779

[56] Leva V, Giuliano S, Bardoni A, Camerini S, Crescenzi M, et al. (2012) Phosphorylation of SRSF1 is modulated by replicational stress. Nucleic acids research 40: 1106–1117. 10.1093/nar/gkr837 21984412 PMC3273819

[57] Xiong Z, Shaibani A, Li YP, Yan Y, Zhang S, et al. (2006) Alternative splicing factor ASF/SF2 is down regulated in inflamed muscle. J Clin Pathol 59: 855–861. 10.1136/jcp.2005.032961 16574722 PMC1860460

