# Supplementary figures and images for: SRSF1 Facilitates Cytosolic DNA-Induced Production of Type I Interferons Recognized by RIG-I
Source: PLoS One. 2015 Feb 6;10(2):e0115354. doi: 10.1371/journal.pone.0115354 (PMC4319963; doi:10.1371/journal.pone.0115354)

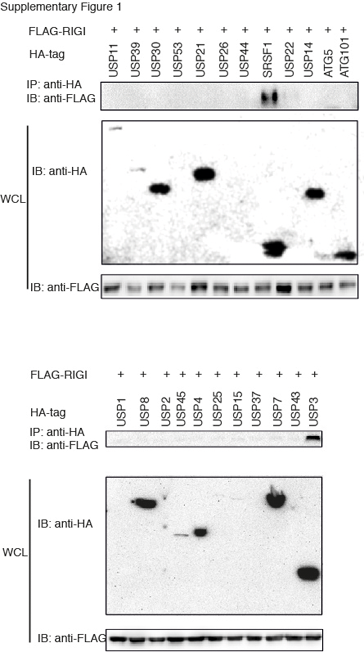

Supplement: S1 Fig — USP family proteins and SRSF1, ATG5, and ATG101 were cloned into pcDNA-HA vectors and cotransfected with Flag-tagged RIG-I into HEK293T cells. Immunoprecipitations were performed using anti-HA beads and immunoblotting was performed using the anti-Flag antibody. (TIF) [file pone.0115354.s001.tif]

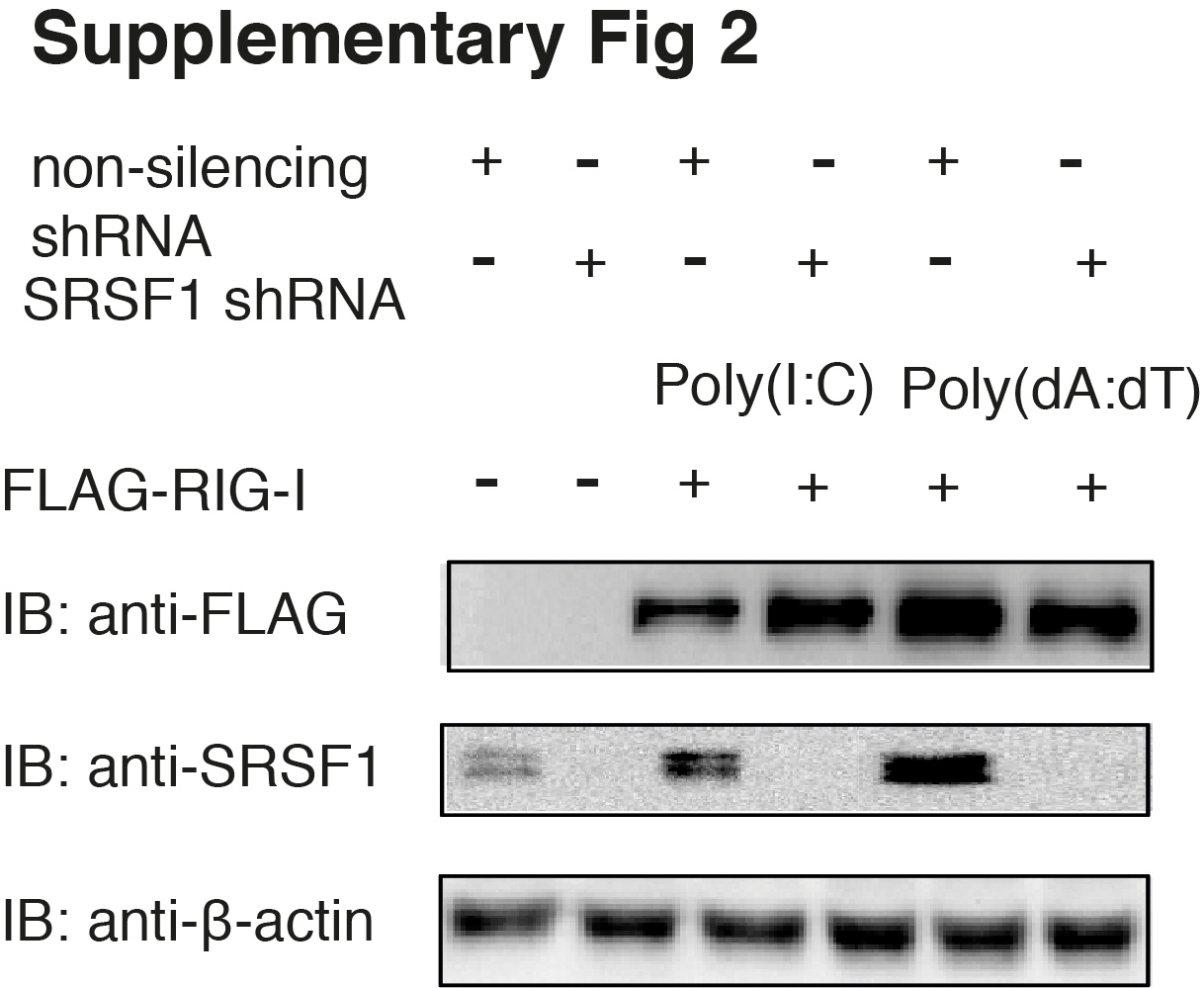

Supplement: S2 Fig — Immunoblot of the samples used in the luciferase assays shown in Fig. 2C using the anti-Flag and anti-SRSF1 antibodies. Actin served as a loading control. (TIF) [file pone.0115354.s002.tif]

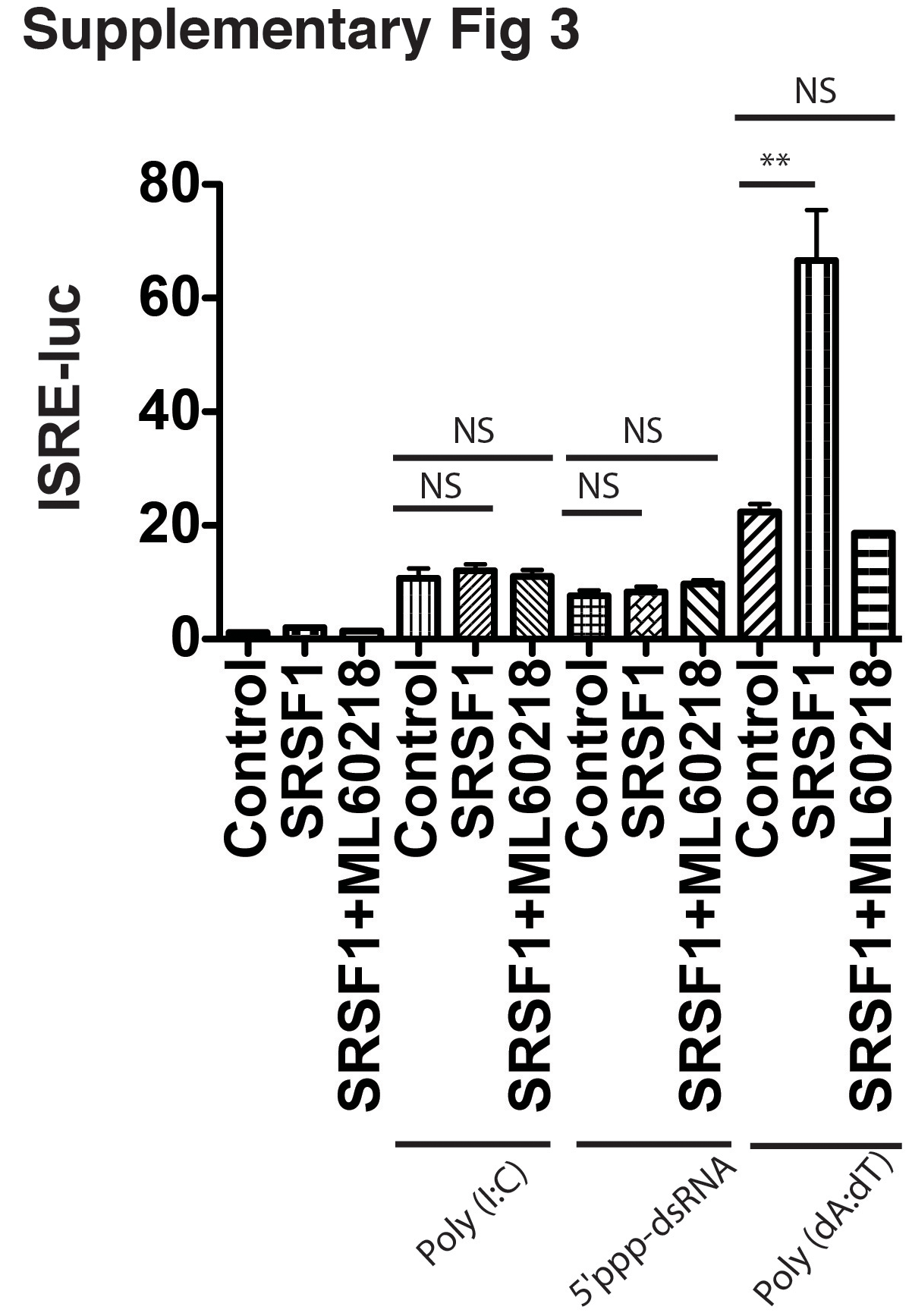

Supplement: S3 Fig — HEK293T cells were treated with ML-60218 (30 μM) or DMSO for 2 h. Cells were subsequently transfected with 0.2 μg/mL poly(dA:dT), 1 μg/mL poly(I:C), or 1 μg/mL 5′ triphosphate double-stranded RNA in conjunction with SRSF1 and ISRE-luciferase reporter plasmids. Luciferase activity was analyzed 24 h after transfection. (TIF) [file pone.0115354.s003.tif]

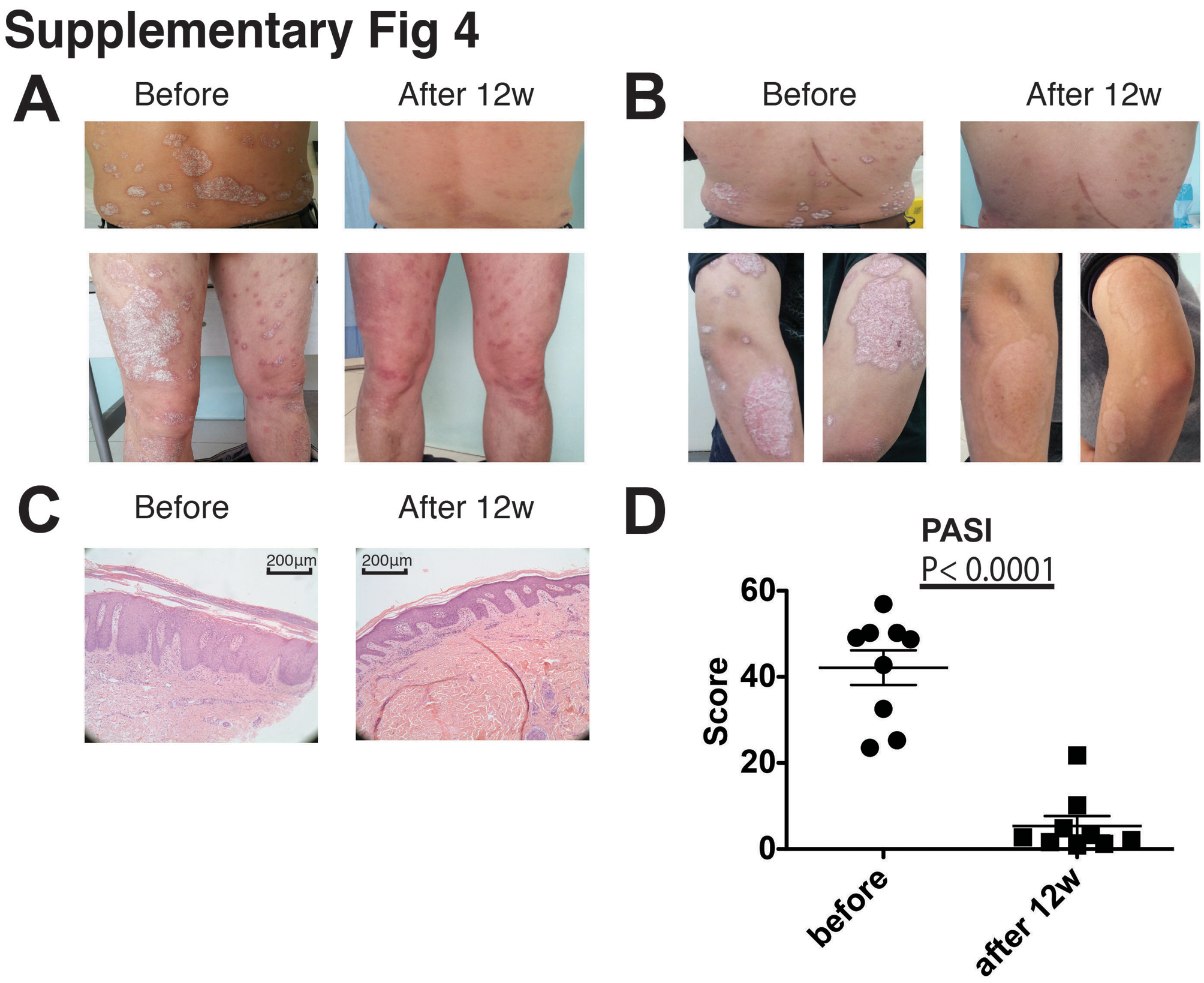

Supplement: S4 Fig — (A, B) Two patients with typical moderate-to-severe chronic plaque psoriasis before and after treatment for 12 weeks with adalimumab are shown. (C) Simultaneous transformations could be observed in patient histopathology. Hyperkeratosis, absence of the granular layer, and epidermal hyperplasia improved after 12 weeks of adalimumab treatment. (D) PASI scores of the nine enrolled patients before and after adalimumab treatment. (TIF) [file pone.0115354.s004.tif]

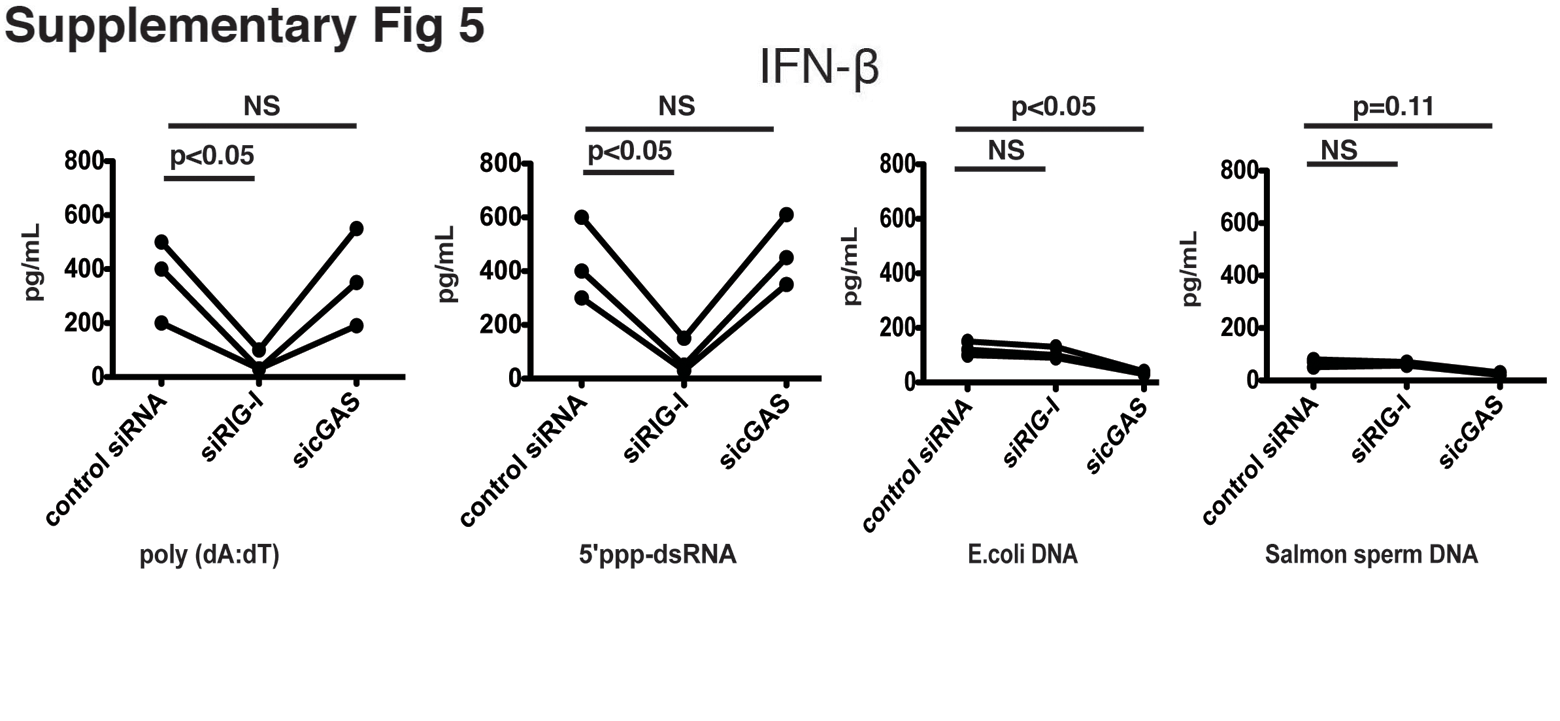

Supplement: S5 Fig — IFN-β concentration in the supernatant of samples stimulated for 24 h with 1 μg/mL poly(dA:dT)/LyoVec or 1ug/mL 5’ppp-dsRNA or 1ug/mL plasmid DNA extracted from E.coli or 1ug/mL sonicated salmon sperm DNAs for 24hrs. Before stimulation, PBMCs from three psoriasis patients before drug treatment were transfected with 300 pmol human RIG-I or cGAS stealth siRNA or scrambled siRNA from invitrogen by electrophoresis and rest for 12 h. (TIF) [file pone.0115354.s005.tif]

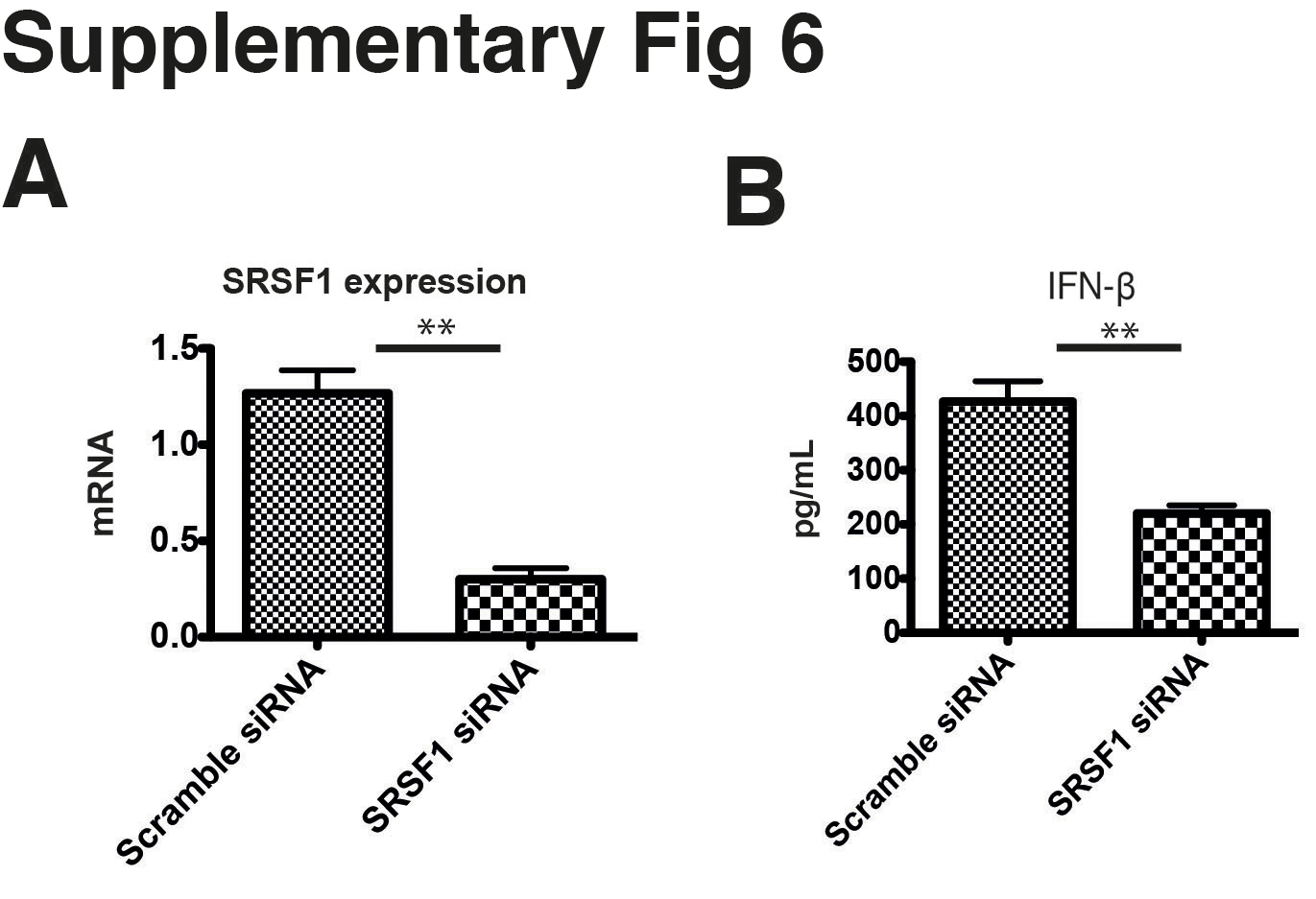

Supplement: S6 Fig — (A) THP-1 cells were electroporated with 300 pmol scrambled siRNA or siRNA targeting human SRSF1. Real-time PCR analyses were used to assess the knockdown efficiency of SRSF1. (B) Thirty-six hours after transfection, THP-1 cells were stimulated for 24 h with 1 μg/mL poly (dA:dT)/LyoVec. Cell supernatants were collected and cytokine levels were measured by ELISA. (TIF) [file pone.0115354.s006.tif]
